# Late-onset versus young-onset rheumatoid arthritis: comorbidities, disease activity, and treatment patterns in a nationwide real-world registry from Kuwait

**DOI:** 10.3389/fmed.2026.1972895

**Published:** 2026-10-08

**Authors:** Anwar Albasri, Ghaydaa Aldabie, Mohammad Khudadah, Ahmad Alsaber, Adeeba Al-Herz, Khuloud Mohammed, Adel Alawadhi, Eman Hasan, Waleed Alkandari, Aqeel Ghanem, Hoda Tarakmeh, Sawsan Hayat, Naser AlHadhood, Ammar Almathkouri, Ali Aldei, Hebah AlHajeri, Fatemah Baron, Amjad Alkadi, Dalal Alkhudir, Yaser Ali

**Affiliations:** 1 Rheumatology Department, Sabah Hospital, Kuwait, Kuwait; 2 Rheumatology Department, Farwaniya Hospital, Kuwait, Kuwait; 3 College of Business and Economics, American University of Kuwait, Kuwait, Kuwait; 4 Rheumatology Department, Amiri Hospital, Kuwait, Kuwait; 5 Department of Medicine, Kuwait University, Kuwait, Kuwait; 6 Rheumatology Department, Mubarak Al-Kabeer Hospital, Kuwait, Kuwait; 7 Rheumatology Department, Adan Hospital, Kuwait, Kuwait; 8 Rheumatology Department, Jaber Hospital, Kuwait, Kuwait

**Keywords:** comorbidity, disease activity, immunosenescence, late-onset rheumatoid arthritis, real-world evidence, registry, rheumatoid arthritis, treat-to-target

## Abstract

**Objectives:**

Late-onset rheumatoid arthritis (LORA) may differ clinically from young-onset rheumatoid arthritis (YORA), but real-world data from the Middle East and North Africa are limited. We compared characteristics, comorbidities, disease activity, and treatment patterns between LORA and YORA in the Kuwait Registry for Rheumatic Diseases (KRRD) and assessed whether age at onset was independently associated with disease activity.

**Methods:**

This retrospective analysis of prospectively collected KRRD data included patients with RA enrolled between January 2012 and April 2026. Patients were classified as YORA (symptom onset 18–59 years) or LORA (≥60 years). Baseline characteristics, disease activity, treatments, and one-year outcomes were compared. Multivariable logistic regression examined factors associated with moderate-to-high disease activity (DAS28-ESR ≥3.2). Sensitivity analyses additionally adjusted for current age, assessed collinearity, and repeated comorbidity and ESR comparisons after age adjustment and within patients aged 60–79 years.

**Results:**

Among 2,629 patients, 303 (11.5%) had LORA and 2,326 (88.5%) had YORA. LORA patients had shorter disease duration (2.9 ± 4.0 vs. 5.0 ± 7.1 years; *p* < 0.001) and higher comorbidity burden (2.0 ± 1.5 vs. 0.9 ± 1.2; *p* < 0.001). Most disease activity measures and treatment patterns were similar. Among 1,402 patients with follow-up of at least one year, disease activity at the last visit was similar (DAS28-ESR mean difference 0.10, 95% CI −0.12 to 0.30), including after inverse-probability weighting and multiple imputation. ESR was higher in LORA (40.9 vs. 33.3 mm/hr; *p* < 0.001), whereas CDAI was not (mean difference 0.7, 95% CI −0.9 to 2.3). Age at onset was not independently associated with moderate-to-high disease activity (adjusted OR 1.08, 95% CI 0.83–1.41; *p* = 0.559). After adjustment for current age and sex, the comorbidity excess in LORA was no longer evident (adjusted rate ratio 0.79, 95% CI 0.70–0.89), while ESR remained modestly higher (adjusted ratio 1.17, 95% CI 1.02–1.33). Adding current age did not materially change the onset-group estimate (adjusted OR 1.05, 95% CI 0.76–1.44).

**Conclusion:**

LORA and YORA showed similar disease activity, treatment patterns, and follow-up outcomes. The higher comorbidity burden in LORA was explained by chronological age, while ESR remained modestly elevated after age adjustment. Findings support treat-to-target principles regardless of age at onset, alongside management of comorbidities in older patients.

## 1 Introduction

Rheumatoid arthritis (RA) remains a primary driver of global disability, characterized by persistent synovitis, progressive joint damage, and functional decline. Despite transformative advances in early diagnosis and therapeutic options, recent Global Burden of Disease data indicate that the absolute prevalence and societal burden of RA continue to increase, largely driven by global population growth and aging (1–3).

This clear shift toward an older patient demographic has fundamentally changed the model of rheumatological practice. Today, we recognize that age at disease onset is an important factor influencing the clinical phenotype, comorbidity profile, and subsequent therapeutic trajectory. Clinically, RA is commonly classified into young-onset rheumatoid arthritis (YORA), with disease onset before 60 years of age, and late-onset rheumatoid arthritis (LORA), with onset ≥60 years of age (4–6). Past cohort studies have shown that LORA often behaves as a distinct entity. It frequently presents with higher inflammatory markers, prominent large-joint involvement, and a higher prevalence of age-related comorbidities (4–7). Nonetheless, the literature remains conflicting regarding how age at onset influences long-term disease activity, treatment adherence, and definitive outcomes.

These clinical differences have a plausible biological basis rather than reflecting chronological age alone. Ageing of the immune system—encompassing immunosenescence and the low-grade systemic inflammatory state termed inflammaging—alters both the magnitude and the character of the synovial inflammatory response, and patients with RA display accelerated immune ageing relative to age-matched individuals without RA (8–10). Late-onset disease is characterized by an intrinsic deficiency of CD8 + regulatory T cells, which leaves synovitis comparatively unchecked, together with an interleukin-6–weighted cytokine profile that contrasts with the tumor necrosis factor–weighted profile predominating in younger-onset disease (11, 12). Because interleukin-6 is the principal driver of the hepatic acute-phase response, this shift is expected to raise the erythrocyte sedimentation rate (ESR) independently of articular disease burden (12, 13). That distinction carries direct therapeutic consequences. ESR is a weighted component of the 28-joint Disease Activity Score using ESR (DAS28-ESR) and rises with age irrespective of synovitis, so ESR-anchored composite indices may overstate inflammatory activity in older patients and prompt treatment escalation that joint counts alone would not support (14, 15). Whether the higher composite scores reported in late-onset cohorts represent genuinely more aggressive synovitis, or partly a measurement property of the index itself, therefore remains unresolved—and bears directly on how disease activity targets should be interpreted at the bedside in older adults.

Managing RA in older patients can be challenging. LORA patients frequently present with multiple overlapping comorbidities, including cardiovascular disease, diabetes mellitus, osteoporosis, and chronic lung disease. These conditions complicate treatment decisions and increase the risk of treatment-related adverse events (5, 6). Comorbidity in RA is not merely a competing clinical concern. Multimorbidity is more prevalent in RA than in matched populations and accumulates more rapidly after diagnosis (16), and a heavier multimorbidity burden has been associated with a lower probability of reaching remission or low disease activity after starting a new disease-modifying antirheumatic drug, even in settings where it does not alter prescribing itself (17, 18). Concerns regarding immunosenescence, polypharmacy, and frailty often contribute to more conservative treatment approaches in older patients (10). Because of this, several real-world registries document significantly lower utilization of conventional and biologic disease-modifying antirheumatic drugs (DMARDs) in LORA cohorts, even when baseline disease severity matches that of younger cohorts (19–21). Whether this trend points to appropriately tailored clinical caution or unnecessary therapeutic conservatism remains a matter of intense debate.

Meanwhile, modern treatment guidelines advocate a strict treat-to-target strategy regardless of patient age, aiming for clinical remission or low disease activity, with drug selection stratified by prognostic factors and by the risk of major adverse cardiovascular events and malignancy rather than by chronological age itself (22). Emerging data suggest that biologic and targeted synthetic therapies retain their efficacy in older cohorts, provided clinicians tailor the treatment to the individual’s specific comorbidity profile rather than managing by chronological age alone (23, 24). Consistent with this, a large international daily-practice cohort found only slight differences in initial treatment choice across onset-age strata and comparable time to the next treatment step, while a treat-to-target strategy trial reported that elderly-onset patients achieved comparable disease activity and patient-reported outcomes over two years despite requiring fewer biologic agents to reach the target (25, 26).

While most comparative studies between YORA and LORA have been conducted in Europe, North America, and East Asia, data from the Gulf region remain limited. This is a gap in the literature that is particularly relevant because Gulf countries have some of the highest rates of obesity, diabetes mellitus, and cardiovascular disease worldwide, factors that may influence both RA presentation and treatment outcomes. The Kuwait Registry for Rheumatic Diseases (KRRD) provides a unique opportunity to evaluate differences between LORA and YORA in a large, real-world Middle Eastern population (27). Regional cohorts from the Gulf report comorbidity and serological profiles that differ appreciably from European and East Asian series, underscoring that observations about age at onset cannot simply be extrapolated across populations (28, 29). Building on the mechanistic considerations above, we tested two linked hypotheses: first, that age at disease onset is not independently associated with disease activity once clinical covariates are accounted for; and second, that any excess in ESR-based disease activity observed in LORA would not be accompanied by a corresponding excess in CDAI, an ESR-independent index, as would be expected if the difference originated in the acute-phase term rather than in articular inflammation. The primary objective of this study was to compare the clinical characteristics, comorbidity burden, disease activity, and treatment patterns at registry enrollment between patients with LORA and YORA. Secondary objectives were to compare one-year disease activity outcomes among patients with available follow-up data and to identify factors independently associated with moderate-to-high disease activity.

## 2 Materials and methods

### 2.1 Study design

This was a retrospective analysis of prospectively collected registry data, comprising a cross-sectional primary comparison at registry enrollment and a longitudinal secondary comparison. Data were extracted from the KRRD in April 2026 and included eligible patients enrolled between January 2012 and the data extraction date. The KRRD is an ongoing multicenter registry that prospectively collects standardized clinical data from patients with rheumatic diseases receiving care across public hospitals in all six governorates of Kuwait. Patients aged 18 years or older who fulfilled the 2010 American College of Rheumatology/European Alliance of Associations for Rheumatology (ACR/EULAR) classification criteria for RA were eligible for inclusion (30). No further exclusion criteria were applied, and all eligible registry records with a documented age at symptom onset were analyzed.

Data collected within the registry include demographic characteristics, comorbidities, laboratory parameters, disease activity measures, and treatment information. Disease activity assessments include the Disease Activity Score in 28 joints using the erythrocyte sedimentation rate (DAS28-ESR) and the Clinical Disease Activity Index (CDAI). Data are prospectively recorded during routine clinical visits approximately every 3–4 months. For the present study, all these variables were obtained from the baseline registry visit. Therefore, these data reflect patient status at the time of registry enrollment and do not necessarily represent disease characteristics at the time of RA diagnosis. Comorbidity burden was defined as the total number of documented comorbid conditions recorded for each patient in the registry at the baseline study visit.

Patients were categorized according to age at disease onset into young-onset rheumatoid arthritis (YORA), defined as disease onset between 18 and 59 years, and late-onset rheumatoid arthritis (LORA), defined as disease onset at or after 60 years of age, consistent with previous studies (4–6). Age at onset was recorded in the registry as the age at which the first RA-attributable symptoms were reported; this variable, and not age at registry enrollment, defined group membership.

Treatment exposure at registry enrollment was categorized as conventional synthetic disease-modifying antirheumatic drugs (csDMARDs), biologic disease-modifying antirheumatic drugs (bDMARDs), targeted synthetic disease-modifying antirheumatic drugs (tsDMARDs), or combination therapy. For regression analyses, advanced therapy was defined as treatment with any bDMARD or tsDMARD, either as monotherapy or in combination with csDMARDs.

The primary analysis consisted of a cross-sectional comparison of demographic characteristics, comorbidity burden, disease activity, and treatment patterns at registry enrollment between patients with LORA and YORA. Secondary analyses evaluated longitudinal outcomes among patients with at least two recorded registry visits and a minimum interval of one year between the baseline and last available visit. For each eligible patient, the last available registry visit was used as the follow-up assessment. Follow-up records of patients without a documented age at onset (*n* = 123), who could not be assigned to an onset group, were excluded, so that the longitudinal sample is fully nested within the baseline cohort. Patients without a subsequent registry visit at least one year after baseline were not included in the longitudinal analysis. DAS28-ESR was selected as the primary outcome measure for these longitudinal analyses because it is a widely validated composite index of rheumatoid arthritis disease activity and was consistently recorded throughout the KRRD. Multivariable logistic regression was performed to identify factors independently associated with moderate-to-high disease activity (DAS28-ESR ≥3.2).

### 2.2 Ethical approval

Ethical approval was obtained from the Joint Committee for the Protection of Human Subjects in Research of the Health Sciences Centre (HSC) and Kuwait Institute for Medical Specialization (KIMS) (2012/VDR/JC/882). The study was conducted in accordance with the ethical principles of the Declaration of Helsinki and its subsequent amendments. All participants provided written informed consent at the time of enrollment. Data extracted from the KRRD were anonymized prior to analysis.

### 2.3 Statistical analysis

Statistical analyses were performed in R (version 4.4.1; R Foundation for Statistical Computing, Vienna, Austria). Continuous variables were presented as mean ± standard deviation (SD) and categorical variables as frequencies and percentages. Comparisons between YORA and LORA were performed using the Wilcoxon rank-sum test for continuous variables and Pearson’s chi-square test or Fisher’s exact test for categorical variables, as appropriate. A two-sided *p*-value <0.05 was considered statistically significant. Means with standard deviations are reported throughout for comparability with previously published LORA–YORA cohorts, while nonparametric tests were used for inference because several continuous variables, including ESR, CRP, and joint counts, were right-skewed. Because a number of the primary comparisons were anticipated to be null, the precision of these comparisons was quantified *post hoc* as the minimum detectable effect size at 80% power and a two-sided alpha of 0.05, given the realized group sizes; this quantifies what magnitude of difference the study could have detected, rather than treating a non-significant result as evidence of equivalence. The primary analyses compared baseline demographic characteristics, comorbidity burden, disease activity, and treatment patterns between LORA and YORA. Secondary analyses evaluated one-year disease activity outcomes among patients with available follow-up data and examined factors independently associated with moderate-to-high disease activity using multivariable logistic regression.

To identify factors associated with moderate-to-high disease activity, multivariable logistic regression analysis was performed using DAS28-ESR ≥3.2 as the dependent variable. Covariates included in the model were age-at-onset group (LORA versus YORA), sex, disease duration, seropositivity status, number of comorbidities, and use of advanced therapy. Model coefficients, standard errors, z-statistics, and corresponding *p*-values were reported. Statistical significance was defined as *p* < 0.05. Covariates were specified *a priori* on clinical grounds rather than by stepwise selection. The model was fitted on complete cases, with patients with missing data for any model covariate excluded from the regression analysis, and the number of complete cases is reported with the model. Model discrimination was assessed by the area under the receiver operating characteristic curve and explanatory performance by McFadden’s pseudo-R²; both are reported so that the strength of the adjusted estimates can be judged directly.

Among patients with at least two registry visits separated by a minimum of one year, disease activity at the last available visit was compared between YORA and LORA. Because follow-up data were not available for the entire cohort, baseline demographic, clinical, and treatment characteristics were additionally compared between patients with and without a one-year assessment, overall and within each onset group, in order to characterize the direction and likely magnitude of attrition bias.

Patients with LORA are older at registry enrollment than patients with YORA, and current age equals age at disease onset plus disease duration. Differences attributed to onset group may thus reflect chronological age. Sensitivity analyses addressed this in four ways. First, we quantified collinearity among current age, age at onset, and disease duration with Pearson correlation coefficients and with variance inflation factors (VIFs) for every covariate in the multivariable model; VIFs below 5 were considered acceptable. Second, we re-estimated the primary logistic regression model for moderate-to-high disease activity with current age (years) added as a continuous covariate, and again with current age replacing the onset-group indicator. Because the onset-group variable is binary, current age and disease duration can be included with it without exact linear dependence. Third, we re-examined the number of comorbidities and ESR, the two baseline measures most likely to vary with chronological age, in models adjusting for current age and sex. Comorbidity count was modeled by Poisson regression with robust (sandwich) standard errors, giving adjusted rate ratios. ESR was log-transformed and modeled by linear regression, giving adjusted geometric-mean ratios; a further model added swollen joint count and CRP to test whether any residual ESR difference tracked articular or acute-phase activity. Fourth, we restricted the cohort to patients currently aged 60–79 years, the range in which both onset groups were adequately represented and repeated the between-group comparisons and the multivariable model within this stratum. Age-matched comparisons are confounded by disease duration in the opposite direction, so these analyses complement rather than replace the primary analysis.

Missing data. Missingness for every analysis variable was tabulated by onset group and compared with Pearson’s *χ*² test (Supplementary Table 3). All percentages and summary statistics in the tables are calculated on patients with non-missing values, and the number with data for each variable is reported in Supplementary Tables 3, 4. The multivariable model was fitted on complete cases as the primary analysis, and patients with complete and incomplete covariate data were compared on baseline characteristics. As a sensitivity analysis, missing covariate and outcome values were imputed by multiple imputation with chained equations (20 imputed datasets; logistic models for binary and linear models for continuous variables, with current age and CDAI as auxiliary variables), the model was refitted in each dataset, and the estimates were pooled by Rubin’s rules.

Effect estimates. Because a non-significant test does not demonstrate equivalence, the principal between-group comparisons are also reported as effect estimates with 95% confidence intervals (Supplementary Table 4): mean differences (LORA minus YORA) with bootstrap percentile intervals from 2,000 resamples, risk differences in percentage points with Newcombe hybrid-score intervals, and adjusted odds ratios from the multivariable model. Odds ratios are expressed for LORA relative to YORA throughout. Intervals were interpreted against the minimal clinically important difference of approximately 0.6 units for DAS28-ESR and of 6 units for CDAI in moderate disease activity; a between-group comparison whose interval excludes these values is described as showing no clinically important difference, and one whose interval does not is described as showing no conclusive difference.

Differential retention. The probability of having a follow-up assessment was modeled by logistic regression on onset group, sex, seropositivity, number of comorbidities, disease duration, advanced therapy, current age, and baseline DAS28-ESR. Three sensitivity analyses used this model (Supplementary Table 5): inverse-probability-of-retention weighting of the follow-up comparison, with bootstrap confidence intervals; multiple imputation of last-visit DAS28-ESR for every patient in the baseline cohort from the same covariates (20 imputations, Rubin’s rules); and a bounding analysis that assigned all non-retained patients in each group to moderate-to-high or to low activity in every combination, with a tipping-point calculation of the proportion of non-retained LORA patients who would need to have moderate-to-high activity for the between-group difference to become statistically significant. In addition, last-visit DAS28-ESR and the odds of DAS28-ESR ≥3.2 and of remission at the last visit were modeled with adjustment for baseline DAS28-ESR, follow-up interval, advanced therapy at enrollment, centre (the three largest hospitals and all others combined), and calendar period of enrollment (2012–2016, 2017–2021, 2022–2026).

Disease duration. Disease duration entered the multivariable model as a continuous covariate. In addition, CDAI, DAS28-ESR, and log-transformed ESR were modeled by linear regression on onset group, disease duration, and sex, and the baseline comparisons were repeated in the subgroup with disease duration of two years or less (Supplementary Table 6). Patients recorded as receiving no DMARD at enrollment were characterized by disease duration, glucocorticoid and NSAID use, and disease activity, and the proportion without DMARD was tabulated by disease-duration category.

## 3 Results

### 3.1 Baseline characteristics

Baseline demographic and clinical characteristics are summarized in Table 1. The study included 2,629 patients with RA, of whom 303 (11.5%) had LORA and 2,326 (88.5%) had YORA. Patients with LORA had a significantly shorter disease duration at registry enrollment than those with YORA (2.9 ± 4.0 vs. 5.0 ± 7.1 years, *p* < 0.001). Seropositivity rates were high and did not differ significantly between the two groups (86.4% vs. 86.0%, *p* = 0.864). Rheumatoid nodules were more common in patients with LORA (5.7% vs. 3.1%, *p* = 0.029), whereas the prevalence of joint deformities was not significantly different between groups (8.9% vs. 6.9%, *p* = 0.233). Missingness did not differ between onset groups for any variable (Supplementary Table 3; all *p* ≥ 0.35); ESR and CRP were missing in 15%–17% of patients in both groups.

**Table 1:** Baseline demographic and clinical characteristics by age at RA onset.

Characteristic	LORA (*n* = 303)	YORA (*n* = 2,326)	Total (*N* = 2,629)	p
Age, M (SD)	68.8 (6.3)	47.3 (11.1)	49.8 (12.7)	<.001
Age at diagnosis, M (SD)	65.5 (4.9)	41.9 (10.7)	44.6 (12.7)	<.001
Male sex, *n* (%)	73 (24.1)	592 (25.5)	665 (25.3)	.609
Current smoker, *n* (%)	28 (12.7)	193 (11.7)	221 (11.9)	.670
BMI, M (SD)	29.6 (5.9)	29.0 (6.0)	29.1 (6.0)	.064
Disease duration, yr, M (SD)	2.9 (4.0)	5.0 (7.1)	4.8 (6.8)	<.001
Seropositive, *n* (%)	241 (86.4)	1,843 (86.0)	2,084 (86.0)	.864
Rheumatoid nodules, *n* (%)	15 (5.7)	62 (3.1)	77 (3.4)	.029
Joint deformities, *n* (%)	24 (8.9)	143 (6.9)	167 (7.1)	.233
Tender joint count, M (SD)	5.6 (8.4)	5.0 (6.7)	5.1 (6.9)	.717
Swollen joint count, M (SD)	1.3 (3.2)	1.7 (3.5)	1.6 (3.5)	.010
Pain VAS, M (SD)	3.0 (2.8)	2.8 (2.7)	2.8 (2.7)	.216
Patient global, M (SD)	3.0 (2.6)	2.7 (2.6)	2.8 (2.6)	.080
ESR, mm/hr, M (SD)	40.9 (26.8)	33.3 (24.0)	34.1 (24.4)	<.001
CRP, mg/L, M (SD)	15.5 (36.8)	12.9 (23.9)	13.2 (25.7)	.483
No. of comorbidities, M (SD)	2.0 (1.5)	0.9 (1.2)	1.0 (1.3)	<.001
*Comorbidities, n (%)*				
Hypertension	154 (50.8)	484 (20.8)	638 (24.3)	<.001
Diabetes mellitus	136 (44.9)	411 (17.7)	547 (20.8)	<.001
Hyperlipidaemia	74 (24.4)	267 (11.5)	341 (13.0)	<.001
Osteoporosis	56 (18.5)	95 (4.1)	151 (5.7)	<.001
Thyroid disease	50 (16.5)	304 (13.1)	354 (13.5)	.100
Asthma	47 (15.5)	220 (9.5)	267 (10.2)	.001
Osteoarthritis	43 (14.2)	161 (6.9)	204 (7.8)	<.001
Coronary artery disease	28 (9.2)	51 (2.2)	79 (3.0)	<.001
Peptic ulcer disease	10 (3.3)	29 (1.2)	39 (1.5)	.011
Interstitial lung disease	7 (2.3)	20 (0.9)	27 (1.0)	.029
Haemoglobinopathy	1 (0.3)	23 (1.0)	24 (0.9)	.514
Hepatitis C	0 (0.0)	13 (0.6)	13 (0.5)	.385
Hepatitis B	1 (0.3)	8 (0.3)	9 (0.3)	>.999

M, mean; SD, standard deviation; BMI, body mass index; VAS, visual analogue scale; ESR, erythrocyte sedimentation rate; CRP, C-reactive protein. Percentages are within onset group. Continuous variables were compared with the Wilcoxon rank-sum test and categorical variables with Pearson’s *χ*² or Fisher’s exact test. Counts reflect non-missing observations; the number of patients with data for each variable is given in Supplementary Table 3.

When compared with YORA, patients with LORA had a significantly greater overall burden of comorbidities (2.0 ± 1.5 versus 0.9 ± 1.2, *p* < 0.001) (Table 1). The prevalence of several comorbid conditions was significantly higher in the LORA group, including hypertension (50.8% versus 20.8%), diabetes mellitus (44.9% versus 17.7%), hyperlipidemia (24.4% versus 11.5%), coronary artery disease (9.2% versus 2.2%), osteoporosis (18.5% versus 4.1%), osteoarthritis (14.2% versus 6.9%), asthma (15.5% versus 9.5%), interstitial lung disease (2.3% versus 0.9%), and peptic ulcer disease (3.3% versus 1.2%) (all *p* < 0.05; Figure 1). The largest absolute differences between groups were observed for hypertension, diabetes mellitus, and osteoporosis. Expressed as effect estimates, patients with LORA had on average 1.11 more comorbid conditions (95% CI 0.94–1.29) and absolute excesses of 30.0 percentage points for hypertension (95% CI 24.2–35.8), 27.2 for diabetes mellitus (21.5–33.0), 14.4 for osteoporosis (10.3–19.2), and 7.0 for coronary artery disease (4.2–10.9) (Supplementary Table 4).

**Figure 1:**
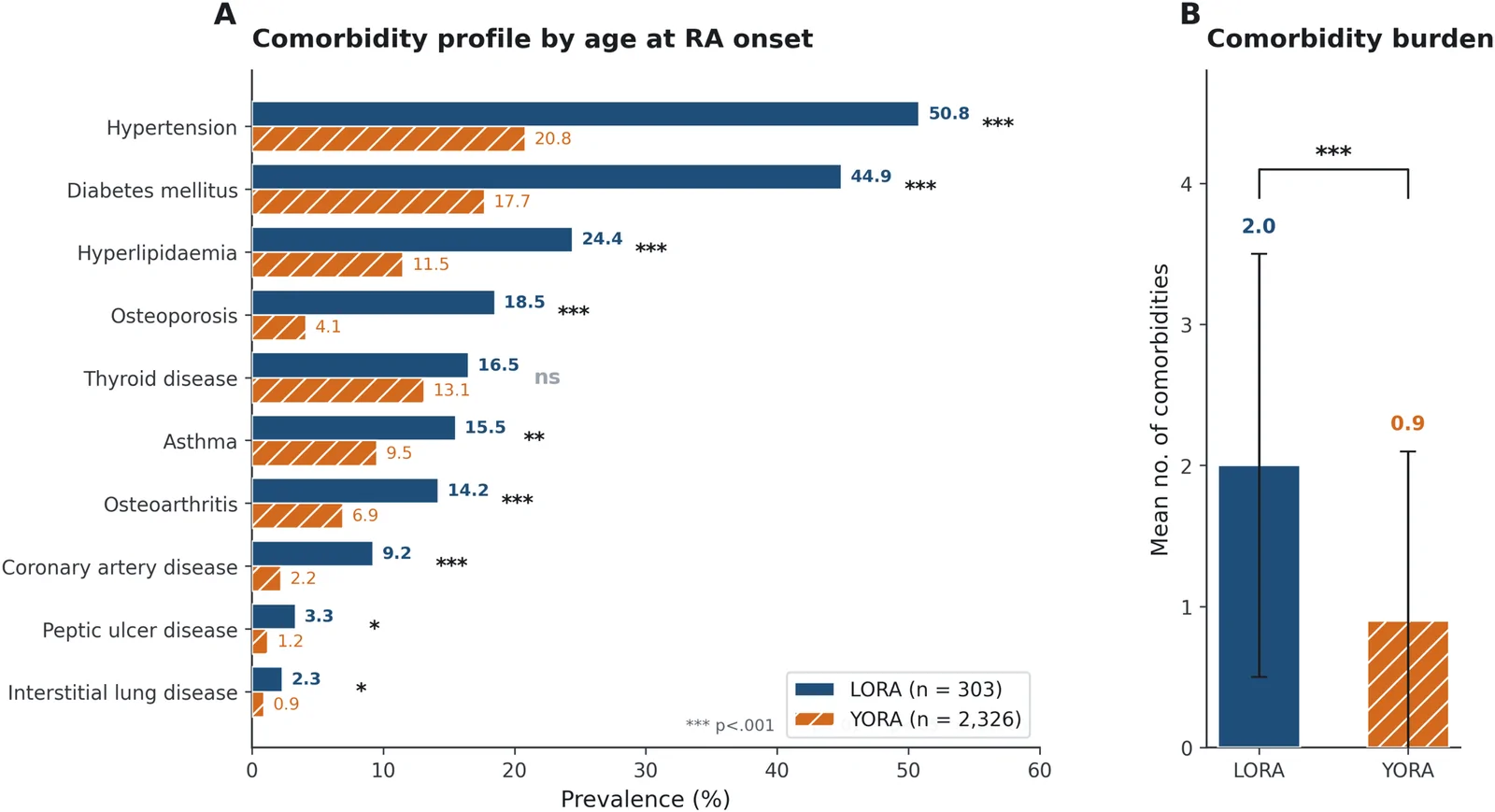
Comorbidity profile and burden in late-onset versus young-onset rheumatoid arthritis. **(A)** Prevalence of selected comorbidities at registry enrollment among patients with late-onset rheumatoid arthritis (LORA) and young-onset rheumatoid arthritis (YORA); values are percentages within onset group. Between-group comparisons used Pearson’s *χ*² test. **(B)** Mean number of comorbidities in the LORA and YORA groups. *p < 0.05, **p < 0.01, ***p < 0.001; ns, not significant. CAD, coronary artery disease; ILD, interstitial lung disease; OA, osteoarthritis.

### 3.2 Baseline disease activity

Baseline inflammatory markers and disease activity measures are summarized in Table 2. ESR was significantly higher in the LORA group compared with the YORA group (40.9 mm/hr vs. 33.3 mm/hr, *p* < 0.001), whereas CRP levels were not significantly different between groups (15.5 mg/L vs. 12.9 mg/L, *p* = 0.483). Tender joint counts, pain scores, and patient and physician global assessments did not differ significantly between groups. Patients with LORA had a modestly lower swollen joint count compared with YORA (1.3 versus 1.7, *p* = 0.010).

**Table 2:** Baseline disease activity by age at RA onset.

Measure	LORA (*n* = 303)	YORA (*n* = 2,326)	Total (*N* = 2,629)	p
CDAI, M (SD)	12.0 (13.7)	11.3 (12.3)	11.4 (12.5)	.592
DAS28-ESR, M (SD)	3.4 (1.5)	3.3 (1.4)	3.3 (1.4)	.105
*DAS28 activity state, n (%)*				.*146*
Remission	95 (31.4)	884 (38.2)	979 (37.4)	
Low	37 (12.2)	258 (11.1)	295 (11.3)	
Moderate	135 (44.6)	918 (39.6)	1,053 (40.2)	
High	36 (11.9)	256 (11.1)	292 (11.1)	
DAS28-ESR ≥ 3.2, *n* (%)	166 (54.8)	1,130 (48.8)	1,296 (49.5)	.050

CDAI, clinical disease activity index; DAS28-ESR, 28-joint Disease Activity Score with erythrocyte sedimentation rate. Continuous measures were compared with the Wilcoxon rank-sum test and categorical measures with Pearson’s *χ*² test. Percentages for the DAS28 activity-state distribution are calculated on non-missing observations (LORA *n* = 303; YORA *n* = 2,316; total *n* = 2,619). Effect estimates with 95% CIs for these comparisons are given in Supplementary Table 4.

No statistically significant between-group differences were observed in CDAI (12.0 versus 11.3; *p* = 0.592) and DAS28 (3.4 versus 3.3; *p* = 0.105). Similarly, the distribution of patients across the DAS28 disease activity states did not differ significantly (*p* = 0.146). The higher ESR in LORA was not accompanied by higher CRP or clinical disease activity measures. Remission rates were 31.4% in LORA and 38.2% in YORA. The proportion of patients with DAS28 ≥ 3.2 was numerically higher in LORA (54.8% vs. 48.8%), although the difference did not reach the prespecified significance threshold (*p* = 0.050) and the comparison was underpowered for a difference of this magnitude, as quantified below. Effect estimates with 95% CIs (Supplementary Table 4) place these findings in context. The mean differences (LORA minus YORA) were 7.6 mm/hr for ESR (95% CI 4.0–11.3), 2.5 mg/L for CRP (−1.8 to 7.6), 0.7 units for CDAI (−0.9 to 2.3), and 0.14 units for DAS28-ESR (−0.04 to 0.30). The intervals for CDAI and DAS28-ESR exclude differences as large as the minimal clinically important difference, whereas the absolute differences in the proportion with DAS28-ESR ≥3.2 (6.0 percentage points, 95% CI 0.0–11.9) and in DAS28-ESR remission (−6.8 percentage points, 95% CI −12.2 to −1.0; *p* = 0.024) were less precisely estimated and are compatible with a modest excess of ESR-based activity in LORA.

### 3.3 Baseline treatment patterns

Baseline treatment patterns are summarized in Table 3. The distribution of treatment strategies, including csDMARD monotherapy, bDMARD monotherapy, combination therapy, and no DMARD use, did not differ significantly between LORA and YORA (*p* = 0.363). Similarly, the distribution of biologic classes did not differ significantly between groups (*p* = 0.582). Overall use of csDMARDs (36.3% versus 39.6%, *p* = 0.276), bDMARDs (20.1% versus 17.3%, *p* = 0.221), and tsDMARDs (0.3% versus 1.0%, *p* = 0.514) also did not differ significantly between groups. The corresponding risk differences (LORA minus YORA) were −3.2 percentage points for csDMARD use (95% CI −8.8 to 2.6), 2.8 for bDMARD use (−1.6 to 7.9), and 0.5 for no DMARD at enrollment (−5.5 to 6.3) (Supplementary Table 4).

**Table 3:** Baseline treatment patterns by age at RA onset.

Variable	LORA (*n* = 303)	YORA (*n* = 2,326)	Total (*N* = 2,629)	p
*Treatment strategy, n (%)*				.*363*
csDMARD monotherapy	74 (24.4)	631 (27.1)	705 (26.8)	
bDMARD monotherapy	25 (8.3)	126 (5.4)	151 (5.7)	
tsDMARD monotherapy	1 (0.3)	8 (0.3)	9 (0.3)	
csDMARD+bDMARD	36 (11.9)	275 (11.8)	311 (11.8)	
csDMARD+tsDMARD	0 (0.0)	14 (0.6)	14 (0.5)	
bDMARD+tsDMARD	0 (0.0)	1 (0.0)	1 (0.0)	
No DMARD	167 (55.1)	1,271 (54.6)	1,438 (54.7)	
*Biologic class, n (%)*				.*582*
Anti-TNF	42 (13.9)	269 (11.6)	311 (11.8)	
Non-anti-TNF	19 (6.3)	129 (5.5)	148 (5.6)	
JAK inhibitor	1 (0.3)	23 (1.0)	24 (0.9)	
Other	0 (0.0)	3 (0.1)	3 (0.1)	
None	241 (79.5)	1,902 (81.8)	2,143 (81.5)	
Any csDMARD use, *n* (%)	110 (36.3)	920 (39.6)	1,030 (39.2)	.276
Any bDMARD use, *n* (%)	61 (20.1)	402 (17.3)	463 (17.6)	.221
Any tsDMARD use, *n* (%)	1 (0.3)	23 (1.0)	24 (0.9)	.514

csDMARD, conventional synthetic disease-modifying antirheumatic drug; bDMARD, biologic DMARD; tsDMARD, targeted synthetic DMARD; TNF, tumor necrosis factor; JAK, Janus kinase. Comparisons used Pearson’s *χ*² or Fisher’s exact test. No comparison was statistically significant.

### 3.4 One-year outcomes

Longitudinal outcomes are summarized in Table 4. A follow-up visit at least one year after enrollment was available for 1,402 patients (146 LORA, 1,256 YORA), all nested within the baseline cohort. Retention was lower in LORA than in YORA (48.2% vs. 54.0%; risk difference −5.8 percentage points, 95% CI −11.7 to 0.2; *p* = 0.056). The interval between the baseline and last visit had a median of 3.5 years (interquartile range 2.1–6.6) and did not differ between groups (LORA 3.6 [2.0–7.2] vs. YORA 3.5 [2.1–6.5] years; *p* = 0.922). At the last visit, mean DAS28-ESR was 2.5 (SD 1.2) in LORA and 2.4 (SD 1.3) in YORA (mean difference 0.10, 95% CI −0.12 to 0.30; *p* = 0.191). Remission was recorded in 57.5% of LORA and 62.5% of YORA patients (risk difference −5.0 percentage points, 95% CI −13.5 to 3.2; *p* = 0.242), and DAS28-ESR <3.2 in 78.8% and 77.0% (risk difference 1.8, 95% CI −5.9 to 8.1; *p* = 0.628); the distribution across activity states did not differ (*p* = 0.159). The within-person change in DAS28-ESR from baseline was −0.80 (SD 1.67) in LORA and −0.78 (SD 1.72) in YORA (difference −0.02, 95% CI −0.32 to 0.26; *p* = 0.950). No conclusive between-group difference in longitudinal disease activity was therefore observed, and the confidence interval for the mean DAS28-ESR difference excludes differences larger than about 0.3 units in either direction.

**Table 4:** Disease activity at one-year follow-up by age at RA onset.

Variable	LORA (*n* = 146)	YORA (*n* = 1,256)	Total (*N* = 1,402)	p
DAS28-ESR, M (SD)	2.5 (1.2)	2.4 (1.3)	2.4 (1.3)	.191
*DAS28 activity state, n (%)*				.*159*
Remission	84 (57.5)	785 (62.5)	869 (62.0)	
Low	31 (21.2)	182 (14.5)	213 (15.2)	
Moderate	25 (17.1)	248 (19.7)	273 (19.5)	
High	6 (4.1)	41 (3.3)	47 (3.4)	
DAS28-ESR < 3.2, *n* (%)	115 (78.8)	967 (77.0)	1,082 (77.2)	.628

DAS28-ESR, 28-joint Disease Activity Score with erythrocyte sedimentation rate. Analysis was restricted to patients in the baseline cohort with a follow-up assessment ≥1 year after enrollment (last available visit; median interval 3.5 years, interquartile range 2.1–6.6). Activity states: remission <2.6; low 2.6 to <3.2; moderate 3.2–5.1; high >5.1. Continuous measures were compared with the Wilcoxon rank-sum test and categorical measures with Pearson’s *χ*² test, consistent with the baseline analyses. Effect estimates with 95% CIs are given in Supplementary Table 4 and sensitivity analyses for differential retention in Supplementary Table 5.

Baseline characteristics of patients with and without a one-year assessment are compared in Supplementary Table 1. Because retention was lower in the LORA group, the longitudinal comparison is conditioned on a subgroup that may not be exchangeable with the full cohort; the one-year estimates are accordingly reported as descriptive rather than as unbiased effect estimates and are interpreted with that constraint in the Discussion.

### 3.5 Factors associated with moderate-to-high disease activity

Factors associated with moderate-to-high disease activity are summarized in Table 5. Multivariable logistic regression analysis was performed to examine factors independently associated with moderate-to-high disease activity (DAS28-ESR ≥3.2) among 2,399 patients with complete data. (230 patients, 8.8% of the cohort, were excluded because of missing covariate data.) The overall model was statistically significant (likelihood ratio *χ*²(6) = 37.0, *p* < 0.001), although its explanatory power was limited (McFadden’s pseudo-R² = 0.011). Male sex (*β* = −0.284, *p* = 0.003) and longer disease duration (*β* = −0.034, *p* < 0.001) were independently associated with lower odds of moderate-to-high disease activity, while seropositivity showed a borderline association with higher disease activity (*β* = 0.214, *p* = 0.072). After adjustment for the other variables in the model, onset group was not independently associated with moderate-to-high disease activity (LORA vs. YORA, adjusted OR 1.08, 95% CI 0.83–1.41; *p* = 0.559), and neither were the number of comorbidities (OR 1.05 per condition, 95% CI 0.98–1.12) or advanced therapy (OR 1.12, 95% CI 0.90–1.39). The model demonstrated limited discriminative performance (AUC = 0.584); therefore, the adjusted associations should be interpreted with caution. To place the null findings in context, the realized group sizes provided 80% power to detect a difference of 0.24 DAS28-ESR units between LORA and YORA, well below the minimal clinically important difference of approximately 0.6 units, but only 8.6 percentage points for the proportion with DAS28-ESR ≥3.2, which exceeds the 6.0-point difference actually observed. These calculations characterize the magnitude of differences detectable with the realized sample sizes but do not establish equivalence; the dichotomized DAS28-ESR comparison was less precise, with an observed between-group difference of 6.0 percentage points. Patients excluded for missing covariate data (*n* = 230), predominantly missing seropositivity status (*n* = 207), had longer disease duration (mean 7.7 vs. 4.5 years; *p* < 0.001), were less often male (18.7% vs. 25.9%; *p* = 0.016), and less often had moderate-to-high activity (41.8% vs. 50.2%; *p* = 0.017) than complete cases, but did not differ in onset group, comorbidity count, or advanced-therapy use (Supplementary Table 3). After multiple imputation of missing covariates and outcome (*n* = 2,629), the estimate for onset group was unchanged (LORA vs. YORA, OR 1.11, 95% CI 0.86–1.43; *p* = 0.440), as were the other coefficients (Supplementary Table 3).

**Table 5:** Multivariable logistic regression of factors associated with moderate-to-high disease activity (DAS28-ESR ≥3.2).

Predictor	b	SE	Z	OR [95% CI]	p
Intercept	−0.027	0.123	−0.221	—	.825
Onset (LORA vs. YORA)	0.080	0.136	0.584	1.08 [0.83, 1.41]	.559
Sex (male vs. female)	−0.284	0.095	−2.983	0.75 [0.62, 0.91]	.003
Seropositive (vs. seronegative)	0.214	0.119	1.797	1.24 [0.98, 1.57]	.072
No. of comorbidities (per unit)	0.046	0.035	1.317	1.05 [0.98, 1.12]	.188
Disease duration (per year)	−0.034	0.007	−4.776	0.97 [0.95, 0.98]	<.001
Advanced therapy (yes vs. no)	0.111	0.112	0.986	1.12 [0.90, 1.39]	.324

Outcome: DAS28-ESR ≥3.2 versus <3.2 (*N* = 2,399 complete cases). Odds ratios are expressed for LORA relative to YORA. b, log-odds coefficient; SE, standard error; OR, odds ratio; CI, confidence interval. Model fit: *χ*²(6) = 37.0, *p* < 0.001; McFadden’s R² = 0.011; AIC = 3,303; deviance = 3,289. Classification at the 0.51 cut-off: accuracy = 54.8%, sensitivity = 55.1%, specificity = 54.6%, AUC = 0.584. Multiple-imputation estimates (*n* = 2,629) are given in Supplementary Table 3.

### 3.6 Sensitivity analyses for confounding by current age

Results of the sensitivity analyses are summarized in Supplementary Table 2. Current age was strongly correlated with age at onset (r = 0.85) but only weakly with disease duration (r = 0.26), and age at onset was weakly inversely correlated with disease duration (r = −0.28). In the multivariable model including current age, all VIFs were below 2 (onset group 1.58; current age 1.92; disease duration 1.29; number of comorbidities 1.34), indicating that collinearity did not compromise the adjusted estimates.

Adding current age to the primary model (*n* = 2,399) left the onset-group estimate essentially unchanged (LORA vs. YORA, adjusted OR 1.05, 95% CI 0.76–1.44; *p* = 0.772), and current age itself was not associated with moderate-to-high disease activity (OR 1.00 per year, 95% CI 0.99–1.01; *p* = 0.710). The estimates for male sex, disease duration, seropositivity, comorbidity count, and advanced therapy were unaltered, and model discrimination was unchanged (AUC = 0.584; McFadden’s pseudo-R² = 0.011). Replacing the onset-group indicator with current age gave the same result (OR 1.00 per year, 95% CI 0.99–1.01; *p* = 0.529).

The comorbidity findings, by contrast, were sensitive to adjustment for current age. In the unadjusted Poisson model, patients with LORA had 2.22 times as many comorbidities as patients with YORA (95% CI 2.01–2.46). After adjustment for current age and sex, this excess was no longer present, and the adjusted rate ratio was below unity (LORA vs. YORA, 0.79, 95% CI 0.70–0.89; *p* < 0.001), with each additional year of current age associated with a 5.4% increase in the comorbidity count (rate ratio 1.054, 95% CI 1.050–1.058). Consistently, among patients currently aged 60–79 years (LORA *n* = 275; YORA *n* = 265; mean age 67.5 vs. 64.5 years), the mean number of comorbidities was 1.9 versus 2.0 (*p* = 0.800), and the prevalences of hypertension (50.9% vs. 49.1%), diabetes mellitus (44.7% vs. 38.9%), osteoporosis (16.4% vs. 16.2%), and coronary artery disease (9.5% vs. 8.3%) did not differ (all *p* > 0.15).

ESR behaved differently. The unadjusted geometric-mean ratio for LORA versus YORA was 1.29 (95% CI 1.16–1.45); after adjustment for current age and sex the ratio was attenuated but remained above unity (1.17, 95% CI 1.02–1.33; *p* = 0.021), and it was further attenuated and no longer statistically significant after additional adjustment for swollen joint count and CRP (1.13, 95% CI 0.98–1.29; *p* = 0.098). Within the age-overlap stratum, ESR remained higher in LORA (40.3 vs. 33.7 mm/hr; *p* = 0.003). In this stratum, however, the age-matched YORA comparators had substantially longer disease duration (12.5 vs. 2.4 years; *p* < 0.001) and, in crude comparisons, lower CDAI (9.4 vs. 11.9; *p* = 0.022) and DAS28-ESR (3.0 vs. 3.4; *p* = 0.003), with a lower proportion in moderate-to-high activity (42.6% vs. 53.8%; *p* = 0.009); swollen joint counts did not differ (1.1 vs. 1.3; *p* = 0.791). When the multivariable model was fitted within the stratum (*n* = 496), the onset-group association was again not statistically significant after adjustment for disease duration and the other covariates (LORA vs. YORA, adjusted OR 1.32, 95% CI 0.84–2.07; *p* = 0.226), illustrating that comparisons at fixed current age are confounded by disease duration just as comparisons at fixed disease duration are confounded by current age.

### 3.7 Sensitivity analyses for missing follow-up

In the retention model (Supplementary Table 5), LORA was associated with a lower, though not statistically significant, probability of having a follow-up assessment after adjustment (OR 0.78, 95% CI 0.57–1.07; *p* = 0.117). Seropositive patients were more likely to be retained (OR 1.42, 95% CI 1.13–1.79; *p* = 0.003) and patients with higher baseline DAS28-ESR less likely (OR 0.92 per unit, 95% CI 0.87–0.98; *p* = 0.006), whereas sex, comorbidity count, disease duration, advanced therapy, and current age were not associated with retention. Weighting the follow-up comparison by the inverse probability of retention (weights 1.57–3.04; *n* = 1,284) left the estimates essentially unchanged: the weighted mean difference in last-visit DAS28-ESR was 0.12 (95% CI −0.10 to 0.34), and the weighted risk differences were −2.0 percentage points for DAS28-ESR ≥3.2 (95% CI −9.6 to 5.5) and −5.2 for remission (−14.4 to 4.0). Multiple imputation of last-visit DAS28-ESR for the full cohort (*n* = 2,626) gave a mean difference of 0.12 (95% CI −0.09 to 0.32) and risk differences of 0.7 (−6.2 to 7.6) and −5.1 (−12.7 to 2.6) percentage points, respectively. The bounding analysis was uninformative at its extremes, as expected with 1,227 non-retained patients (proportion with DAS28-ESR ≥3.2 ranging from 10.2% to 62.0% in LORA and from 12.4% to 58.4% in YORA), but the tipping-point calculation showed that the observed null difference would become statistically significant if 35% or more of the 157 non-retained LORA patients had moderate-to-high activity while non-retained YORA patients resembled those retained (23.0%). The longitudinal null finding is therefore robust to retention that depends on measured baseline characteristics, but not to strongly differential selection on unmeasured factors. In models adjusting for baseline DAS28-ESR, follow-up interval, advanced therapy, centre, and calendar period of enrollment (*n* = 1,398), the LORA coefficient for last-visit DAS28-ESR was −0.01 (95% CI −0.22 to 0.20; *p* = 0.946), the adjusted OR for DAS28-ESR ≥3.2 was 0.78 (95% CI 0.51–1.21; *p* = 0.272), and the adjusted OR for remission was 0.91 (95% CI 0.63–1.31; *p* = 0.620). Baseline DAS28-ESR (0.17 units per unit; *p* < 0.001) and follow-up interval (−0.03 units per year; *p* = 0.004) were the only covariates associated with last-visit disease activity.

### 3.8 Disease duration and patients without DMARD therapy at enrollment

Because patients with LORA had a shorter disease duration at enrollment, the baseline comparisons were repeated with adjustment for disease duration and within early disease (Supplementary Table 6). After adjustment for disease duration and sex, the differences (LORA minus YORA) were 0.13 units for CDAI (95% CI −1.37 to 1.63), and 0.08 for DAS28-ESR (−0.09 to 0.25), whereas the geometric-mean ratio for ESR was 1.28 (95% CI 1.15–1.44). Among 1,385 patients with disease duration of two years or less (196 LORA, 1,189 YORA), CDAI (12.7 vs. 13.3; *p* = 0.272), DAS28-ESR (3.50 vs. 3.49; *p* = 0.949), and the proportion with DAS28-ESR ≥3.2 (57.7% vs. 55.4%; *p* = 0.559) were similar, ESR remained higher in LORA (41.8 vs. 34.3 mm/hr; *p* < 0.001), swollen joint counts were lower (1.5 vs. 2.2; *p* = 0.002), and the comorbidity difference persisted (1.8 vs. 0.7; *p* < 0.001). In this early-disease subgroup, bDMARD use was more frequent in LORA (15.8% vs. 7.4%; *p* < 0.001), and the proportion without DMARD did not differ (66.8% vs. 72.4%; *p* = 0.109).

Of the 1,438 patients (54.7%) recorded as receiving no DMARD at enrollment, the proportion was the same in LORA and YORA (55.1% vs. 54.6%). These patients had a median disease duration of 1.0 year (interquartile range 0–4) compared with 5.0 years among DMARD-treated patients; 48.0% had been diagnosed less than one year before enrollment and 69.0% within two years. The proportion without DMARD fell steeply with disease duration, from 78.4% among the 880 patients diagnosed less than one year earlier to 63.5% at one to two years, 49.7% at two to five years, and 32.3% at five years or more. Among patients without DMARD, 26.3% were receiving glucocorticoids and 36.4% an NSAID at enrollment, and disease activity was higher than in DMARD-treated patients (mean DAS28-ESR 3.38 vs. 3.15, *p* < 0.001; CDAI 12.1 vs. 10.6, *p* < 0.001; DAS28-ESR ≥3.2 in 51.8% vs. 46.7%). This profile is that of recently diagnosed, active disease captured at registry entry before DMARD initiation rather than of untreated established disease. Because the medication fields in the analytic extract are carried forward from the enrollment visit, subsequent DMARD initiation in this group could not be quantified.

## 4 Discussion

The principal finding of this nationwide registry study is that patients with LORA had a substantially greater burden of comorbidities than those with young-onset disease. Despite this, most observed disease activity measures, treatment patterns, and one-year outcomes among patients with available follow-up did not differ significantly between the two groups. In the adjusted analysis, no statistically significant independent association was observed between age-at-onset group and moderate-to-high disease activity. This finding should be interpreted in the context of the model’s limited explanatory and discriminative performance. Read together with the divergence between ESR and the other disease activity measures, these findings suggest that the higher ESR observed in LORA may partly reflect the influence of ESR on the measurement instrument rather than greater articular disease activity. However, greater underlying inflammatory activity cannot be excluded. Overall, our results suggest that older age at disease onset should not by itself modify the treat-to-target approach, and that the clinically consequential difference between LORA and YORA in this population is one of comorbidity load rather than of articular disease activity. Sensitivity analyses adjusting for current age indicated that this comorbidity difference was attributable to the older chronological age of patients with LORA rather than to age at onset itself, whereas the null association between onset group and disease activity was robust to age adjustment.

The higher rate of comorbidities observed in our study among LORA patients is consistent with previous reports. Innala et al. and Tan et al. similarly reported higher rates of cardiovascular and metabolic comorbidities among patients with LORA compared with YORA (5, 6). In our cohort, the differences were particularly marked for hypertension, diabetes mellitus, coronary artery disease, osteoporosis, and hyperlipidemia. These findings are clinically relevant because comorbidities often influence treatment decisions and complicate long-term disease management. Given the high prevalence of diabetes mellitus, obesity, and cardiovascular disease in Gulf countries, the burden of comorbidity observed in our LORA cohort is not unexpected. The age-adjusted sensitivity analyses qualify this finding in an important way. Once current age and sex were taken into account, the comorbidity excess in LORA disappeared, and among patients in the overlapping age range of 60–79 years the comorbidity count and the prevalences of hypertension, diabetes mellitus, osteoporosis, and coronary artery disease did not differ between onset groups. The greater comorbidity burden of LORA in this cohort therefore reflects the chronological age at which these patients present rather than a property of late-onset disease itself. This is consistent with the observation that multimorbidity in RA accumulates with age in a manner that parallels, but exceeds, that of the general population (16). The distinction matters for interpretation but not for practice: irrespective of whether the burden is attributable to onset type or to age, the rheumatologist who diagnoses RA in a patient aged 60 years or older is confronted, from the first visit, with a comorbidity load roughly twice that of the typical younger-onset patient, and the case for structured comorbidity screening at diagnosis is unchanged.

It is therefore notable that comorbidity count was not independently associated with moderate-to-high disease activity in our adjusted model. This contrasts with registry data from the United States, in which a heavier multimorbidity burden predicted a lower likelihood of reaching remission or low disease activity after initiating a new disease-modifying antirheumatic drug (17, 18). Several explanations are plausible. Our comorbidity measure was a simple count, which neither weights conditions by severity nor captures the interconnectedness central to the concept of multimorbidity; the conditions predominating in our cohort were metabolic and cardiovascular rather than the mental-health and chronic-pain patterns most strongly linked to disease activity elsewhere (18); and disease activity here was assessed cross-sectionally at enrollment rather than as a response to a treatment change. The absence of an association in our data should therefore not be read as evidence that comorbidity is inconsequential to RA outcomes, but rather as an indication that a crude count is an insensitive instrument for detecting that relationship.

Even with greater comorbidity burden and higher ESR levels among patients with LORA, most disease activity measures did not differ significantly between groups. We found no statistically significant differences in CDAI, DAS28, and remission rate, although there was a borderline difference in the proportion of patients with DAS28 ≥ 3.2 at baseline. Previous studies have reported conflicting findings regarding disease severity in LORA, with some suggesting a more inflammatory phenotype and others reporting outcomes comparable to those in YORA (4–6, 31, 32). Our findings align with reports in which disease activity at registry enrollment did not differ conclusively between LORA and YORA despite the greater burden of comorbidities observed in the older-onset group. In addition, seropositivity rates were high and did not differ significantly between groups. This differs from some previous studies that reported lower seropositivity among patients with LORA (33). The confidence intervals sharpen this statement. Differences as large as the minimal clinically important difference were excluded for CDAI (upper limit 2.3 units against a threshold of 6 34) and for DAS28-ESR (upper limit 0.30 units against a threshold of 0.6), whereas the intervals for the proportions in moderate-to-high activity and in remission were compatible with an excess of ESR-based activity in LORA of up to about 12 percentage points. We therefore describe these findings as the absence of a conclusive difference rather than as equivalence, and we have avoided the term comparable disease control.

The shorter disease duration of LORA at enrollment (2.9 vs. 5.0 years) means that the baseline comparison contrasts groups at somewhat different stages of disease, and disease duration was itself the strongest correlate of disease activity in the multivariable model, with lower odds of moderate-to-high activity for each additional year. Comparing LORA with YORA at enrollment therefore tends, if anything, to favor YORA, whose disease was on average more established and more often already treated. Three observations indicate that this did not drive the findings. Disease duration was adjusted for in the multivariable model; linear models adjusting for duration and sex gave between-group differences in CDAI and DAS28-ESR that were close to zero with narrow intervals; and within early disease, where the two groups were at comparable stages, the disease activity indices were again similar while the ESR and comorbidity differences persisted. Within early disease, moreover, patients with LORA were about twice as likely as those with YORA to be receiving a bDMARD (15.8% vs. 7.4%), which argues further against therapeutic conservatism toward older patients in this setting.

The pattern of these results is itself informative. ESR was substantially higher in LORA, while CRP and CDAI did not differ between groups. ESR rises with age through mechanisms independent of synovitis, including age-related increases in plasma fibrinogen and immunoglobulin concentrations, and the interleukin-6–weighted cytokine milieu of late-onset disease amplifies this effect (11, 12). Because ESR enters DAS28-ESR with substantial weight, differences in ESR may influence the composite score even when clinical measures of articular disease activity are similar, and the same mechanism has been shown to produce systematic misclassification of DAS28-ESR disease activity states across other patient strata (14, 15). Interpreted in this light, the borderline excess of DAS28-ESR ≥3.2 in LORA may partly reflect age-related influences on ESR; however, this remains a plausible explanation, and greater underlying inflammatory activity cannot be excluded from these data. The practical implication is that ESR-based measures should be interpreted alongside clinical assessment and ESR-independent indices such as CDAI when applying treat-to-target thresholds in older patients whose ESR is elevated out of proportion to their joint counts. The age-adjusted analyses are informative here as well. ESR rises with age in healthy adults, largely through age-related increases in fibrinogen and immunoglobulin concentrations, and is higher in women than in men at any age (35, 36). In our data the ESR excess in LORA was attenuated by roughly half after adjustment for current age and sex but did not disappear (adjusted ratio 1.17), and it was further attenuated and no longer statistically significant once swollen joint count and CRP were also taken into account. This pattern suggests that the higher ESR in LORA is the product of two components, an age-related shift in the ESR baseline and a modest excess in acute-phase activity, neither of which is captured by the swollen joint count. It reinforces the practical point that ESR-anchored composite scores should be read alongside CDAI, an ESR-independent index, in older patients, while cautioning against attributing the entire ESR difference to a measurement artifact.

A second finding of note was the absence of major differences in treatment patterns between LORA and YORA. Previous studies have reported lower utilization of biologic therapies among older patients despite comparable disease activity (19–21). In contrast, we observed no statistically significant between-group differences in the use of csDMARDs, bDMARDs, or tsDMARDs. These findings suggest that rheumatologists participating in the KRRD adopted similar treatment strategies regardless of age at disease onset, despite the greater burden of comorbidities among patients with LORA. While our study design does not allow us to determine the clinical factors underlying individual treatment decisions, the findings do not suggest systematic therapeutic conservatism in routine clinical practice across participating centers. This is consistent with the METEOR international daily-practice cohort, in which initial treatment differed only slightly across onset-age strata and time to a subsequent treatment step was similar (25), and with the tREACH strategy trial, in which elderly-onset patients reached comparable targets despite lower biologic exposure (26). A plausible structural explanation in the Kuwaiti setting is that rheumatology care and advanced therapies are publicly funded and delivered through a small number of government hospitals operating under shared protocols, which removes the cost and access gradients that elsewhere tend to track with patient age. The high proportion of patients recorded as receiving no DMARD at enrollment (54.7%) may reflect registry entry occurring at or shortly after diagnosis, before therapy is established, rather than with untreated prevalent disease. The data support this interpretation. Nearly half of the untreated patients had been diagnosed within the preceding year, the proportion without DMARD fell from 78% among those diagnosed less than a year earlier to 32% among those with disease of five years or more, and untreated patients had higher disease activity and more glucocorticoid use than DMARD-treated patients, the profile of newly referred active disease awaiting DMARD initiation rather than of treatment gaps. Access barriers are an unlikely explanation, because csDMARDs and advanced therapies are provided without charge through the public hospitals that contribute to the registry, and the proportion without DMARD was identical in the two onset groups. Whether these patients started DMARD therapy at subsequent visits could not be quantified, because the analytic extract carries enrollment medications forward, and this remains a limitation of the treatment data.

Our longitudinal analysis, in which the last available visit occurred a median of 3.5 years after enrollment, reinforces the observation at baseline. Disease activity scores, remission rates, and the proportion of patients with DAS28-ESR ≥3.2 did not differ significantly between LORA and YORA. This observation is consistent with previous studies demonstrating that biologic and targeted therapies remain effective in appropriately selected older patients (23, 24). However, because these longitudinal analyses were limited to patients with available follow-up, and because retention was appreciably lower in the LORA group (48.2% versus 54.0%), the one-year findings are susceptible to differential attrition. Were patients lost to follow-up to differ systematically from those retained—for instance if older patients with poorly controlled disease were more likely to transfer care, be hospitalized, or die— observed between-group differences at one year would be biased toward the null. These estimates are therefore best regarded as hypothesis-generating. The sensitivity analyses temper but do not remove this concern. After adjustment, retention depended on seropositivity and baseline disease activity rather than on onset group, and neither inverse-probability weighting nor multiple imputation of the last-visit DAS28-ESR changed the estimates. The tipping-point analysis showed, however, that the null finding would be overturned if roughly one third or more of the non-retained LORA patients had moderate-to-high activity, a scenario that cannot be excluded because the registry does not record reasons for non-attendance. The longitudinal finding is thus robust to selection on measured characteristics only.

In the multivariable analysis, age-at-onset group was not independently associated with moderate-to-high disease activity after adjustment for relevant clinical factors. Male sex (OR 0.75, 95% CI 0.62–0.91) and longer disease duration (OR 0.97 per year, 95% CI 0.95–0.98) were associated with lower odds of DAS28-ESR ≥3.2, whereas seropositivity showed a borderline association. Given the limited explanatory and discriminative performance of the model, these findings should be interpreted with appropriate caution. Equally important, however, is what the interval excludes. The adjusted OR for LORA relative to YORA was 1.08 (95% CI 0.83–1.41), and the multiple-imputation estimate was 1.11 (95% CI 0.86–1.43). This interval is compatible with odds in LORA ranging from 17% lower to 41% higher than in YORA and therefore should not be interpreted as demonstrating equivalence. The low pseudo-R² and modest AUC indicate that the measured covariates explain little of the between-patient variation in disease activity, which is unsurprising when the dominant determinants—cumulative treatment intensity, adherence, and unmeasured patient-level factors—are not captured in a cross-sectional model; residual confounding therefore remains possible.

The strengths of this study include the use of a large nationwide registry, representation of routine clinical practice across multiple centers, and the availability of one-year follow-up data. Coverage is close to national, since the registry draws on the rheumatology services of public hospitals in all six governorates, which limits the referral bias affecting single-center comparisons of onset age, and the simultaneous availability of CDAI and DAS28-ESR permitted the index-dependence of the findings to be examined directly. Several limitations should be acknowledged. First, the study was based on registry data and is therefore subject to the limitations inherent to observational research, including residual confounding and potential selection bias. Because LORA and YORA are inherently associated with differences in current age, residual confounding by age-related factors cannot be fully excluded. Sensitivity analyses adjusting for current age, and comparisons restricted to the age range in which both groups overlapped, were undertaken to address this concern; they showed that the onset-group estimate for disease activity was robust to age adjustment, whereas the comorbidity difference was not. Nevertheless, because current age equals age at onset plus disease duration, the three quantities cannot be disentangled simultaneously in any observational design, and each adjusted estimate is conditional on the other two. Age-matched comparisons in particular contrast recent-onset LORA with long-standing YORA, so that residual confounding by disease duration, cumulative treatment exposure, and survivor effects cannot be excluded. Moreover, imaging measures of synovitis and age-adjusted inflammatory assessments were not available; therefore, we could not determine whether the higher ESR in LORA reflected age-related influences on ESR or differences in underlying inflammatory activity. In addition, age at disease onset was based on the reported age at first RA-attributable symptoms and may therefore be subject to recall error. Second, disease activity and treatment variables were assessed at registry enrollment rather than at the time of RA diagnosis. Consequently, the study does not provide information regarding differences in disease presentation or initial treatment strategies between LORA and YORA. The long study period also spanned changes in RA treatment practices, and potential temporal and center-level effects could not be fully accounted for. Medication data reflected recorded treatment exposure and did not capture cumulative treatment history, dose, duration, adherence, or reasons for treatment changes, and the analytic extract carried enrollment medications forward to subsequent visits, so that treatment at follow-up could not be evaluated. Third, 46.7% of the cohort lacked a follow-up assessment at least one year after enrollment. Consequently, the longitudinal analyses may be susceptible to attrition bias if patients with and without follow-up differed systematically, and these findings should therefore be interpreted cautiously. Loss to follow-up was moreover differential by onset group, so the longitudinal comparison rests on a subgroup that is not fully exchangeable with the source cohort. Retention was lower in LORA than in YORA (48.2% vs. 54.0%). The registry does not systematically record reasons for absence of subsequent follow-up; therefore, we could not determine whether differential retention was related to mortality, transfer of care, discontinuation of follow-up, or other factors. The sensitivity analyses indicate that the longitudinal estimates are robust to retention that depends on measured baseline characteristics, but the tipping-point analysis shows that they are not robust to strongly differential selection on unmeasured factors, and the direction of any such bias cannot be determined from the available data. Follow-up timing was not uniform because the last available visit occurring at least one year after baseline was used, which may have introduced additional variability in the longitudinal comparisons. Fourth, although current glucocorticoid use is captured at baseline in the registry, cumulative glucocorticoid exposure over time is not, and information on frailty, functional status, radiographic progression, socioeconomic factors, and patient-reported outcomes was unavailable; residual confounding by these factors cannot be excluded. Missing covariate data, chiefly seropositivity status, resulted in exclusion of 230 patients (8.8%) from the complete-case multivariable analysis; excluded patients had longer disease duration and lower disease activity, but multiple imputation gave estimates indistinguishable from the complete-case results. Fifth, DAS28-ESR was selected as the longitudinal outcome because it was recorded most consistently across the registry, yet it is precisely the index most vulnerable to the age-related acute-phase effect discussed above, and CDAI-based longitudinal analyses were not feasible with the available follow-up data. The study was not designed as an equivalence or non-inferiority study, and equivalence margins were not prespecified; therefore, non-significant between-group comparisons should not be interpreted as evidence of equivalence. Finally, causal inferences cannot be drawn from the observed associations.

From a clinical perspective, our findings suggest that older age at RA onset alone should not preclude the use of treat-to-target strategies or appropriate advanced therapies. Rather, treatment decisions should be individualized, taking into account comorbidities, functional status, and overall treatment goals. Two practical implications follow. First, the findings are consistent with applying treat-to-target principles irrespective of age at disease onset, while considering the greater comorbidity burden in LORA. Second, when ESR is elevated out of proportion to clinical findings in an older patient, ESR-based disease activity measures should be interpreted alongside clinical assessments and ESR-independent indices such as CDAI. The corollary is that the clinical work specific to late-onset disease lies less in modifying the rheumatological target than in the parallel management of a comorbidity burden more than twice that of younger-onset patients, where structured comorbidity screening at the time of RA diagnosis is likely to yield the greatest return (16). Future prospective studies incorporating frailty, radiographic progression, glucocorticoid exposure, and patient-reported outcomes are warranted to better define long-term outcomes and optimize management strategies for patients with LORA.

## 5 Conclusion

In this nationwide registry, patients with LORA had a substantially greater burden of comorbidities but no statistically significant differences in most disease activity measures or treatment patterns at registry enrollment compared with YORA. Moderate-to-high DAS28-ESR activity was numerically more frequent in LORA, alongside higher ESR, whereas CDAI did not differ significantly. Among patients with a follow-up assessment, disease activity at the last visit did not differ conclusively between groups, and this finding was unchanged after weighting and imputation for differential retention. These findings suggest that age at disease onset alone should not be the sole determinant of treatment decisions, although these observational findings do not establish equivalence or causality, and the longitudinal findings should be interpreted cautiously given differential attrition and variable follow-up timing. The excess inflammatory signal observed in LORA was confined to the ESR term and was not reflected in CDAI, which argues for preferring ESR-independent indices when applying treat-to-target thresholds in older adults. Future prospective studies incorporating frailty, functional status, glucocorticoid exposure, radiographic progression, and patient-reported outcomes are needed to better define long-term outcomes in older adults with rheumatoid arthritis. Age-adjusted sensitivity analyses showed that the comorbidity excess was accounted for by the older chronological age of patients with LORA, whereas the absence of an independent association between onset group and disease activity was robust to adjustment for current age.

## Data Availability

The raw data supporting the conclusions of this article will be made available by the authors, without undue reservation.
