# Supplementary material for: Late-onset versus young-onset rheumatoid arthritis: comorbidities, disease activity, and treatment patterns in a nationwide real-world registry from Kuwait
Source: Front Med (Lausanne). 2026 Oct 8;13:1972895. doi: 10.3389/fmed.2026.1972895 (PMC13686842; doi:10.3389/fmed.2026.1972895)
Supplement: Supplementary file 1 [file Table1.docx]

**Supplementary**

Supplementary Table 1 (revised). Baseline Characteristics of Patients With and Without a Follow-Up Assessment ≥1 Year After Enrollment, Overall and by Age at RA Onset

| **A. Whole cohort (N = 2,629)** | **Retained (n = 1,402)** | **Not retained (n = 1,227)** | **p** |
| --- | --- | --- | --- |
| Current age, years, M (SD) | 49.5 (12.1) | 50.0 (13.2) | .427 |
| Disease duration, years, M (SD) | 4.9 (7.0) | 4.7 (6.6) | .729 |
| BMI, M (SD) | 29.1 (5.8) | 29.1 (6.3) | .672 |
| No. of comorbidities, M (SD) | 1.0 (1.3) | 1.0 (1.2) | .852 |
| ESR, mm/hr, M (SD) | 33.6 (23.4) | 34.8 (25.6) | .581 |
| CRP, mg/L, M (SD) | 13.8 (28.5) | 12.7 (22.2) | .012 |
| CDAI, M (SD) | 10.2 (11.8) | 12.8 (13.1) | < .001 |
| DAS28-ESR, M (SD) | 3.2 (1.4) | 3.4 (1.5) | .006 |
| Male sex, n (%) | 365 (26.0) | 300 (24.4) | .351 |
| Seropositive, n (%) | 1138 (87.9) | 946 (83.9) | .005 |
| Current smoker, n (%) | 97 (10.2) | 124 (13.6) | .025 |
| DAS28-ESR ≥ 3.2, n (%) | 655 (46.8) | 641 (52.6) | .003 |
| Any csDMARD use, n (%) | 595 (42.4) | 435 (35.5) | < .001 |
| Advanced therapy, n (%) | 256 (18.3) | 230 (18.7) | .749 |
| Hypertension, n (%) | 317 (22.6) | 321 (26.2) | .034 |
| Diabetes mellitus, n (%) | 280 (20.0) | 267 (21.8) | .260 |
| LORA, n (%) | 146 (10.4) | 157 (12.8) | .056 |
| **B. LORA (n = 303)** | **Retained (n = 146)** | **Not retained (n = 157)** | **p** |
| Current age, years, M (SD) | 68.0 (5.7) | 69.5 (6.7) | .079 |
| Disease duration, years, M (SD) | 2.6 (3.7) | 3.3 (4.2) | .171 |
| BMI, M (SD) | 29.1 (4.9) | 30.2 (6.7) | .334 |
| No. of comorbidities, M (SD) | 2.0 (1.5) | 2.0 (1.5) | .644 |
| ESR, mm/hr, M (SD) | 40.7 (25.4) | 41.1 (28.4) | .727 |
| CRP, mg/L, M (SD) | 15.0 (40.9) | 15.9 (32.9) | .737 |
| CDAI, M (SD) | 9.9 (12.0) | 14.0 (14.8) | .017 |
| DAS28-ESR, M (SD) | 3.3 (1.3) | 3.5 (1.6) | .408 |
| Male sex, n (%) | 35 (24.0) | 38 (24.2) | .962 |
| Seropositive, n (%) | 114 (86.4) | 127 (86.4) | .994 |
| Current smoker, n (%) | 9 (8.9) | 19 (16.0) | .118 |
| DAS28-ESR ≥ 3.2, n (%) | 72 (49.3) | 94 (59.9) | .065 |
| Any csDMARD use, n (%) | 58 (39.7) | 52 (33.1) | .232 |
| Advanced therapy, n (%) | 34 (23.3) | 28 (17.8) | .240 |
| Hypertension, n (%) | 63 (43.2) | 91 (58.0) | .010 |
| Diabetes mellitus, n (%) | 56 (38.4) | 80 (51.0) | .028 |
| **C. YORA (n = 2,326)** | **Retained (n = 1,256)** | **Not retained (n = 1,070)** | **p** |
| Current age, years, M (SD) | 47.4 (10.8) | 47.2 (11.4) | .694 |
| Disease duration, years, M (SD) | 5.1 (7.2) | 4.9 (6.8) | .511 |
| BMI, M (SD) | 29.1 (5.9) | 28.9 (6.2) | .394 |
| No. of comorbidities, M (SD) | 0.9 (1.3) | 0.9 (1.1) | .584 |
| ESR, mm/hr, M (SD) | 32.7 (23.0) | 34.0 (25.1) | .562 |
| CRP, mg/L, M (SD) | 13.6 (26.7) | 12.2 (20.0) | .010 |
| CDAI, M (SD) | 10.2 (11.7) | 12.7 (12.9) | < .001 |
| DAS28-ESR, M (SD) | 3.2 (1.4) | 3.3 (1.5) | .010 |
| Male sex, n (%) | 330 (26.3) | 262 (24.5) | .324 |
| Seropositive, n (%) | 1024 (88.0) | 819 (83.6) | .003 |
| Current smoker, n (%) | 88 (10.4) | 105 (13.2) | .074 |
| DAS28-ESR ≥ 3.2, n (%) | 583 (46.5) | 547 (51.5) | .016 |
| Any csDMARD use, n (%) | 537 (42.8) | 383 (35.8) | < .001 |
| Advanced therapy, n (%) | 222 (17.7) | 202 (18.9) | .454 |
| Hypertension, n (%) | 254 (20.2) | 230 (21.5) | .451 |
| Diabetes mellitus, n (%) | 224 (17.8) | 187 (17.5) | .822 |

Note. Retained = patients with a follow-up visit ≥1 year after enrollment within the baseline cohort (n = 1,402); not retained = all other patients in the baseline cohort (n = 1,227). Continuous variables compared with the Wilcoxon rank-sum test and categorical variables with Pearson's χ² test; percentages calculated on non-missing observations. M = mean; SD = standard deviation; BMI = body mass index; ESR = erythrocyte sedimentation rate; CRP = C-reactive protein; CDAI = Clinical Disease Activity Index; DAS28-ESR = 28-joint Disease Activity Score with ESR; csDMARD = conventional synthetic disease-modifying antirheumatic drug. This table replaces the previously submitted Supplementary Table 1, which was derived from a follow-up sample that included 123 records not belonging to the baseline cohort.

Supplementary Table 2. Sensitivity Analyses Addressing Confounding by Current Age

| **Analysis** | **Comparison** | **n** | **Estimate** | **95% CI** | **p** |
| --- | --- | --- | --- | --- | --- |
| **A. Collinearity diagnostics** |  |  |  |  |  |
| Current age vs. age at onset | Pearson r | 2,626 | 0.85 | — | — |
| Current age vs. disease duration | Pearson r | 2,612 | 0.26 | — | — |
| Age at onset vs. disease duration | Pearson r | 2,612 | −0.28 | — | — |
| VIF, Model 2 | Onset group | 2,399 | 1.58 | — | — |
|  | Current age |  | 1.92 | — | — |
|  | Disease duration |  | 1.29 | — | — |
|  | No. of comorbidities |  | 1.34 | — | — |
|  | Sex / seropositivity / advanced therapy |  | 1.03 / 1.01 / 1.11 | — | — |
| **B. Moderate-to-high disease activity (DAS28-ESR ≥ 3.2), logistic regression** |  |  |  |  |  |
| Model 1 (primary; Table 5) | LORA vs. YORA | 2,399 | OR 1.08 | 0.83–1.41 | .559 |
| Model 2 (Model 1 + current age) | LORA vs. YORA | 2,399 | OR 1.05 | 0.76–1.44 | .772 |
|  | Current age, per year |  | OR 1.002 | 0.993–1.011 | .710 |
| Model 3 (current age in place of onset group) | Current age, per year | 2,399 | OR 1.002 | 0.995–1.010 | .529 |
| Model 4 (Model 1 within age-overlap stratum) | LORA vs. YORA | 496 | OR 1.32 | 0.84–2.07 | .226 |
| **C. Number of comorbidities, Poisson regression** |  |  |  |  |  |
| Unadjusted | LORA vs. YORA | 2,626 | RR 2.22 | 2.01–2.46 | < .001 |
| Adjusted for current age and sex | LORA vs. YORA | 2,626 | RR 0.79 | 0.70–0.89 | < .001 |
|  | Current age, per year |  | RR 1.054 | 1.050–1.058 | < .001 |
| **D. ESR, linear regression of log(ESR)** |  |  |  |  |  |
| Unadjusted | LORA vs. YORA | 2,213 | Ratio 1.29 | 1.16–1.45 | < .001 |
| Adjusted for current age and sex | LORA vs. YORA | 2,213 | Ratio 1.17 | 1.02–1.33 | .021 |
| Additionally adjusted for SJC and CRP | LORA vs. YORA | 1,820 | Ratio 1.13 | 0.98–1.29 | .098 |
| **E. Age-overlap stratum, current age 60–79 years (LORA n = 275; YORA n = 265)** |  |  |  |  |  |
| Current age, years, M | LORA vs. YORA | 275 / 265 | 67.5 vs. 64.5 | — | < .001 |
| Disease duration, years, M | LORA vs. YORA | 274 / 265 | 2.4 vs. 12.5 | — | < .001 |
| No. of comorbidities, M | LORA vs. YORA | 275 / 265 | 1.9 vs. 2.0 | — | .800 |
| Hypertension, % | LORA vs. YORA | 275 / 265 | 50.9 vs. 49.1 | — | .667 |
| Diabetes mellitus, % | LORA vs. YORA | 275 / 265 | 44.7 vs. 38.9 | — | .168 |
| Osteoporosis, % | LORA vs. YORA | 275 / 265 | 16.4 vs. 16.2 | — | .966 |
| Coronary artery disease, % | LORA vs. YORA | 275 / 265 | 9.5 vs. 8.3 | — | .638 |
| ESR, mm/hr, M | LORA vs. YORA | 228 / 230 | 40.3 vs. 33.7 | — | .003 |
| CRP, mg/L, M | LORA vs. YORA | 234 / 214 | 16.1 vs. 10.6 | — | .059 |
| Swollen joint count, M | LORA vs. YORA | 275 / 265 | 1.3 vs. 1.1 | — | .791 |
| CDAI, M | LORA vs. YORA | 275 / 265 | 11.9 vs. 9.4 | — | .022 |
| DAS28-ESR, M | LORA vs. YORA | 275 / 264 | 3.4 vs. 3.0 | — | .003 |
| DAS28-ESR ≥ 3.2, % | LORA vs. YORA | 275 / 265 | 53.8 vs. 42.6 | — | .009 |

Note. VIF = variance inflation factor; OR = odds ratio; RR = rate ratio; CI = confidence interval; ESR = erythrocyte sedimentation rate; CRP = C-reactive protein; CDAI = Clinical Disease Activity Index; DAS28-ESR = 28-joint Disease Activity Score with ESR; SJC = swollen joint count. Model 1 is the primary model reported in Table 5. Poisson models used robust (sandwich) standard errors. ESR models were fitted on log-transformed ESR; estimates are geometric-mean ratios. The age-overlap stratum comprised patients currently aged 60–79 years; continuous variables were compared with the Wilcoxon rank-sum test and proportions with Pearson's χ² test. — = not applicable.

Supplementary Table 3. Missing Data by Onset Group, Comparison of Complete and Incomplete Cases, and Multiple-Imputation Estimates

| **Variable** | **LORA / complete** | **YORA / incomplete** | **n with data** | **p** |
| --- | --- | --- | --- | --- |
| **A. Missing values by onset group** |  |  |  |  |
| Current age | 1 (0.3) | 2 (0.1) | 2,626 | .237 |
| BMI | 95 (31.4) | 724 (31.1) | 1,810 | .936 |
| Current smoking | 83 (27.4) | 682 (29.3) | 1,864 | .487 |
| Disease duration | 3 (1.0) | 14 (0.6) | 2,612 | .428 |
| Seropositivity | 24 (7.9) | 183 (7.9) | 2,422 | .974 |
| Rheumatoid nodules | 39 (12.9) | 322 (13.8) | 2,268 | .644 |
| Joint deformities | 33 (10.9) | 254 (10.9) | 2,342 | .988 |
| Tender joint count | 0 (0.0) | 0 (0.0) | 2,629 | — |
| Swollen joint count | 0 (0.0) | 0 (0.0) | 2,629 | — |
| Pain VAS | 0 (0.0) | 2 (0.1) | 2,627 | .610 |
| Patient global | 0 (0.0) | 2 (0.1) | 2,627 | .610 |
| ESR | 51 (16.8) | 362 (15.6) | 2,216 | .568 |
| CRP | 45 (14.9) | 399 (17.2) | 2,185 | .314 |
| CDAI | 0 (0.0) | 3 (0.1) | 2,626 | .532 |
| DAS28-ESR | 0 (0.0) | 10 (0.4) | 2,619 | .253 |
| No. of comorbidities | 0 (0.0) | 0 (0.0) | 2,629 | — |
| Treatment strategy | 0 (0.0) | 0 (0.0) | 2,629 | — |
| **B. Complete (n = 2,399) vs. incomplete (n = 230) cases for the multivariable model** |  |  |  |  |
| LORA, % | 11.5 | 11.7 | 2,399 / 230 | .915 |
| Male sex, % | 25.9 | 18.7 | 2,399 / 230 | .016 |
| Current age, years, M | 49.7 | 50.1 | 2,399 / 227 | .567 |
| Disease duration, years, M | 4.5 | 7.7 | 2,399 / 213 | < .001 |
| Seropositive, % | 86.0 | 87.0 | 2,399 / 23 | .899 |
| No. of comorbidities, M | 1.0 | 1.0 | 2,399 / 230 | .753 |
| CDAI, M | 11.6 | 9.5 | 2,396 / 230 | .042 |
| DAS28-ESR ≥ 3.2, % | 50.2 | 41.8 | 2,399 / 220 | .017 |
| Advanced therapy, % | 18.3 | 20.9 | 2,399 / 230 | .330 |
| **C. Multiple-imputation estimates for the multivariable model (n = 2,629; 20 imputations)** |  |  |  |  |
| LORA vs. YORA | OR 1.11 | 0.86–1.43 | FMI 0.00 | .440 |
| Male vs. female | OR 0.76 | 0.63–0.91 | FMI 0.00 | .003 |
| Seropositive vs. seronegative | OR 1.23 | 0.98–1.56 | FMI 0.07 | .075 |
| No. of comorbidities, per condition | OR 1.06 | 1.00–1.13 | FMI 0.00 | .063 |
| Disease duration, per year | OR 0.97 | 0.95–0.98 | FMI 0.01 | < .001 |
| Advanced therapy, yes vs. no | OR 1.10 | 0.89–1.35 | FMI 0.00 | .383 |

Note. Panel A: n (%) of patients with missing values in each onset group (LORA n = 303; YORA n = 2,326); the fourth column is the number of patients with data, on which the percentages and summary statistics in Tables 1–3 are based; p from Pearson's χ² test. Treatment strategy was recorded for all patients ("no DMARD" is a recorded category, not missing data). Panel B: complete cases are patients with non-missing values for all model variables; columns give complete and incomplete cases, the numbers with data, and p from the Wilcoxon rank-sum or Pearson's χ² test. Panel C: pooled odds ratios (LORA vs. YORA and other covariates) after multiple imputation by chained equations with Rubin's rules; FMI = fraction of missing information. VAS = visual analogue scale; ESR = erythrocyte sedimentation rate; CRP = C-reactive protein; CDAI = Clinical Disease Activity Index; DAS28-ESR = 28-joint Disease Activity Score with ESR; OR = odds ratio.

Supplementary Table 4. Effect Estimates With 95% Confidence Intervals for the Principal Between-Group Comparisons

| **Comparison** | **Measure** | **LORA n** | **YORA n** | **Estimate (95% CI)** |
| --- | --- | --- | --- | --- |
| **A. Baseline comparisons (LORA minus YORA)** |  |  |  |  |
| Disease duration, years | Mean difference | 300 | 2,312 | −2.10 (−2.60 to −1.55) |
| No. of comorbidities | Mean difference | 303 | 2,326 | 1.11 (0.94 to 1.29) |
| Tender joint count | Mean difference | 303 | 2,326 | 0.63 (−0.32 to 1.61) |
| Swollen joint count | Mean difference | 303 | 2,326 | −0.40 (−0.79 to −0.00) |
| Patient global, 0–10 | Mean difference | 303 | 2,324 | 0.26 (−0.06 to 0.57) |
| ESR, mm/hr | Mean difference | 252 | 1,964 | 7.64 (4.04 to 11.27) |
| CRP, mg/L | Mean difference | 258 | 1,927 | 2.52 (−1.83 to 7.64) |
| CDAI | Mean difference | 303 | 2,323 | 0.69 (−0.94 to 2.26) |
| DAS28-ESR | Mean difference | 303 | 2,316 | 0.14 (−0.04 to 0.30) |
| Seropositive | Risk difference, % points | 279 | 2,143 | 0.38 (−4.36 to 4.23) |
| Rheumatoid nodules | Risk difference, % points | 264 | 2,004 | 2.59 (0.22 to 6.13) |
| Hypertension | Risk difference, % points | 303 | 2,326 | 30.02 (24.16 to 35.83) |
| Diabetes mellitus | Risk difference, % points | 303 | 2,326 | 27.21 (21.48 to 33.04) |
| Osteoporosis | Risk difference, % points | 303 | 2,326 | 14.40 (10.33 to 19.21) |
| Coronary artery disease | Risk difference, % points | 303 | 2,326 | 7.05 (4.20 to 10.87) |
| DAS28-ESR remission (<2.6) | Risk difference, % points | 303 | 2,316 | −6.82 (−12.17 to −1.04) |
| DAS28-ESR ≥ 3.2 | Risk difference, % points | 303 | 2,316 | 5.99 (0.01 to 11.87) |
| Any csDMARD use | Risk difference, % points | 303 | 2,326 | −3.25 (−8.83 to 2.64) |
| Any bDMARD use | Risk difference, % points | 303 | 2,326 | 2.85 (−1.57 to 7.95) |
| Any tsDMARD use | Risk difference, % points | 303 | 2,326 | −0.66 (−1.22 to 0.89) |
| Advanced therapy (b/tsDMARD) | Risk difference, % points | 303 | 2,326 | 2.23 (−2.23 to 7.36) |
| No DMARD at enrollment | Risk difference, % points | 303 | 2,326 | 0.47 (−5.51 to 6.34) |
| **B. Longitudinal comparisons (LORA minus YORA)** |  |  |  |  |
| DAS28-ESR at last visit | Mean difference | 146 | 1,256 | 0.10 (−0.12 to 0.30) |
| Change in DAS28-ESR from baseline | Mean difference | 146 | 1,254 | −0.02 (−0.32 to 0.26) |
| Remission (<2.6) at last visit | Risk difference, % points | 146 | 1,256 | −4.97 (−13.49 to 3.22) |
| DAS28-ESR < 3.2 at last visit | Risk difference, % points | 146 | 1,256 | 1.78 (−5.89 to 8.10) |
| Retention with ≥1-year follow-up | Risk difference, % points | 303 | 2,326 | −5.81 (−11.74 to 0.16) |
| **C. Multivariable model** |  |  |  |  |
| Moderate-to-high activity (DAS28-ESR ≥ 3.2), LORA vs. YORA | Adjusted odds ratio | 2,399 |  | 1.08 (0.83 to 1.41) |

Note. Mean differences are LORA minus YORA with bootstrap percentile 95% CIs (2,000 resamples); risk differences are in percentage points with Newcombe hybrid-score 95% CIs; the odds ratio is from the complete-case multivariable model in Table 5. n columns give the numbers of LORA and YORA patients with data for each comparison. Longitudinal comparisons are restricted to the 1,402 patients with a follow-up assessment ≥1 year after enrollment. For reference, the minimal clinically important difference is approximately 0.6 units for DAS28-ESR and 6 units for CDAI in moderate disease activity [36]. VAS = visual analogue scale; ESR = erythrocyte sedimentation rate; CRP = C-reactive protein; CDAI = Clinical Disease Activity Index; DAS28-ESR = 28-joint Disease Activity Score with ESR; csDMARD/bDMARD/tsDMARD = conventional synthetic/biologic/targeted synthetic disease-modifying antirheumatic drug.

Supplementary Table 5. Sensitivity Analyses for Differential Retention in the Longitudinal Analysis

| **Analysis** | **Estimate** | **95% CI** | **p** |
| --- | --- | --- | --- |
| **A. Retention model: probability of a follow-up assessment (n = 2,399)** |  |  |  |
| LORA vs. YORA | OR 0.78 | 0.57–1.07 | .117 |
| Male vs. female | OR 1.12 | 0.93–1.35 | .233 |
| Seropositive vs. seronegative | OR 1.42 | 1.13–1.79 | .003 |
| No. of comorbidities, per condition | OR 1.02 | 0.95–1.10 | .512 |
| Disease duration, per year | OR 1.00 | 0.98–1.01 | .686 |
| Advanced therapy, yes vs. no | OR 0.96 | 0.77–1.20 | .746 |
| Current age, per year | OR 1.00 | 0.99–1.01 | .644 |
| Baseline DAS28-ESR, per unit | OR 0.92 | 0.87–0.98 | .006 |
| **B. Between-group differences at the last visit (LORA minus YORA)** |  |  |  |
| DAS28-ESR, mean difference: unweighted (n = 1,402) | 0.11 | — |  |
| DAS28-ESR, mean difference: inverse-probability weighted (n = 1,284) | 0.12 | −0.10 to 0.34 |  |
| DAS28-ESR, mean difference: multiple imputation, full cohort (n = 2,626) | 0.12 | −0.09 to 0.32 |  |
| DAS28-ESR ≥ 3.2, risk difference (% points): unweighted (n = 1,402) | −2.0 | — |  |
| DAS28-ESR ≥ 3.2, risk difference (% points): inverse-probability weighted (n = 1,284) | −2.0 | −9.6 to 5.5 |  |
| DAS28-ESR ≥ 3.2, risk difference (% points): multiple imputation, full cohort (n = 2,626) | 0.7 | −6.2 to 7.6 |  |
| Remission, risk difference (% points): unweighted (n = 1,402) | −5.5 | — |  |
| Remission, risk difference (% points): inverse-probability weighted (n = 1,284) | −5.2 | −14.4 to 4.0 |  |
| Remission, risk difference (% points): multiple imputation, full cohort (n = 2,626) | −5.1 | −12.7 to 2.6 |  |
| **C. Bounding analysis for DAS28-ESR ≥ 3.2 at the last visit (non-retained: LORA n = 157; YORA n = 1,070)** |  |  |  |
| Observed among retained patients | LORA 21.2% | YORA 23.0% |  |
| All non-retained assigned to moderate-to-high activity | LORA 62.0% | YORA 58.4% |  |
| All non-retained assigned to low activity or remission | LORA 10.2% | YORA 12.4% |  |
| Non-retained LORA active, non-retained YORA inactive (worst case for LORA) | LORA 62.0% | YORA 12.4% |  |
| Non-retained LORA inactive, non-retained YORA active (best case for LORA) | LORA 10.2% | YORA 58.4% |  |
| Tipping point: proportion of non-retained LORA with moderate-to-high activity at which p < 0.05 | 35% | (non-retained YORA at 23.0%) |  |
| **D. Models adjusted for baseline DAS28-ESR, follow-up interval, advanced therapy, centre, and calendar period (n = 1,398)** |  |  |  |
| Last-visit DAS28-ESR, LORA coefficient | −0.01 | −0.22 to 0.20 | .946 |
| DAS28-ESR ≥ 3.2 at last visit, LORA vs. YORA | OR 0.78 | 0.51–1.21 | .272 |
| Remission at last visit, LORA vs. YORA | OR 0.91 | 0.63–1.31 | .620 |

Note. Panel A: logistic regression of having a follow-up assessment ≥1 year after enrollment on baseline characteristics (complete cases). Panel B: differences at the last visit, unweighted, weighted by the inverse of the modeled probability of retention (bootstrap percentile 95% CIs, 2,000 resamples), and after multiple imputation of last-visit DAS28-ESR for all patients in the baseline cohort from the retention-model covariates (20 imputations, Rubin's rules). Panel C: proportion with DAS28-ESR ≥ 3.2 at the last visit under extreme assumptions about non-retained patients; the tipping point is the smallest proportion of non-retained LORA patients with moderate-to-high activity, with non-retained YORA patients at the observed YORA rate, at which the between-group difference becomes statistically significant. OR = odds ratio; DAS28-ESR = 28-joint Disease Activity Score with erythrocyte sedimentation rate. Panel D: linear regression of last-visit DAS28-ESR, and logistic regression of DAS28-ESR ≥ 3.2 and of remission at the last visit, each on onset group, baseline DAS28-ESR, follow-up interval (years), advanced therapy at enrollment, centre (Amiri, Farwaniya, Mubarak Al-Kabeer, other), and calendar period of enrollment (2012–2016, 2017–2021, 2022–2026).

Supplementary Table 6. Analyses Accounting for Disease Duration and Characterization of Patients Without DMARD Therapy at Enrollment

| **Variable** | **LORA / No DMARD** | **YORA / DMARD-treated** | **p** |
| --- | --- | --- | --- |
| **A. Linear models adjusting for disease duration and sex (LORA minus YORA)** |  |  |  |
| CDAI, units (n = 2,609) | 0.13 | −1.37 to 1.63 | .864 |
| DAS28-ESR, units (n = 2,602) | 0.08 | −0.09 to 0.25 | .344 |
| ESR, geometric-mean ratio (n = 2,200) | 1.28 | 1.15–1.44 | < .001 |
| **B. Early disease (duration ≤ 2 years): LORA (n = 196) vs. YORA (n = 1,189)** |  |  |  |
| Current age, years, M | 66.6 (n = 196) | 44.4 (n = 1,189) | < .001 |
| Disease duration, years, M | 0.60 (n = 196) | 0.50 (n = 1,189) | .098 |
| No. of comorbidities, M | 1.8 (n = 196) | 0.7 (n = 1,189) | < .001 |
| Tender joint count, M | 5.9 (n = 196) | 6.1 (n = 1,189) | .198 |
| Swollen joint count, M | 1.5 (n = 196) | 2.2 (n = 1,189) | .002 |
| ESR, mm/hr, M | 41.8 (n = 168) | 34.3 (n = 1,013) | < .001 |
| CRP, mg/L, M | 16.7 (n = 165) | 15.0 (n = 979) | .336 |
| CDAI, M | 12.7 (n = 196) | 13.3 (n = 1,187) | .272 |
| DAS28-ESR, M | 3.50 (n = 196) | 3.49 (n = 1,182) | .949 |
| DAS28-ESR remission, % | 26.0 | 32.9 | .055 |
| DAS28-ESR ≥ 3.2, % | 57.7 | 55.4 | .559 |
| Any csDMARD use, % | 25.5 | 24.5 | .755 |
| Any bDMARD use, % | 15.8 | 7.4 | < .001 |
| Advanced therapy, % | 15.8 | 7.8 | < .001 |
| No DMARD at enrollment, % | 66.8 | 72.4 | .109 |
| **C. Patients recorded as receiving no DMARD at enrollment** | **No DMARD** | **DMARD-treated** |  |
| n (% of cohort) | 1,438 (54.7) | 1,191 (45.3) |  |
| LORA / YORA, % without DMARD | 55.1 / 54.6 | — | .925 |
| Disease duration, years, median (IQR) | 1.0 (0–4) | 5.0 (1–10) |  |
| Diagnosed < 1 year before enrollment, % | 48.0 | 16.0 |  |
| Current glucocorticoid use, % | 26.3 | 21.7 |  |
| Current NSAID use, % | 36.4 | — |  |
| DAS28-ESR, M | 3.38 | 3.15 | < .001 |
| CDAI, M | 12.1 | 10.6 | < .001 |
| DAS28-ESR ≥ 3.2, % | 51.8 | 46.7 |  |
| **D. Proportion without DMARD by disease duration** | **n** | **% without DMARD** |  |
| Duration <1 years | 880 | 78.4 |  |
| Duration 1–<2 years | 301 | 63.5 |  |
| Duration 2–<5 years | 517 | 49.7 |  |
| Duration ≥5 years | 914 | 32.3 |  |

Note. Panel A: coefficients (LORA minus YORA) from linear regression of each index on onset group, disease duration (years), and sex; ESR was log-transformed and the coefficient is expressed as a ratio. Panel B: comparisons restricted to patients with disease duration ≤ 2 years; continuous variables compared with the Wilcoxon rank-sum test and proportions with Pearson's χ² test. Panel C: characteristics of patients recorded as receiving no csDMARD, bDMARD, or tsDMARD at enrollment; glucocorticoid use is calculated on patients with non-missing data. Panel D: proportion without DMARD by disease-duration category. M = mean; IQR = interquartile range; ESR = erythrocyte sedimentation rate; CRP = C-reactive protein; CDAI = Clinical Disease Activity Index; DAS28-ESR = 28-joint Disease Activity Score with ESR; DMARD = disease-modifying antirheumatic drug; NSAID = non-steroidal anti-inflammatory drug.
